# Pathogens Associated with Domestic Cats (*Felis catus*), Their Public Health Impact on Children, and Implications of Urban Management

**DOI:** 10.3390/pathogens15070673

**Published:** 2026-06-25

**Authors:** Reuven Yosef

**Affiliations:** Eilat Campus, Ben Gurion University of the Negev, P.O. Box 272, Eilat 881012, Israel; ryosef60@gmail.com

**Keywords:** *Felis catus*, zoonoses, *Toxoplasma gondii*, *Bartonella henselae*, *Toxocara cati*, children, One Health, Trap–Neuter–Return, urban ecology

## Abstract

Domestic cats (*Felis catus*) are ubiquitous companion animals that provide substantial psychological and social benefits to children and adults alike, but they also serve as reservoirs and vectors for a wide range of zoonotic pathogens. Close physical contact between cats and children, frequent use of shared environments such as homes, playgrounds, and sandboxes, and still-developing hygiene behaviours increase opportunities for exposure to protozoa, helminths, bacteria, fungi, and ectoparasite-borne agents. This review synthesizes current evidence on key feline-associated zoonoses of pediatric concern—including *Toxoplasma gondii*, *Toxocara cati*, *Ancylostoma* spp., *Dipylidium caninum*, *Bartonella henselae*, *Salmonella enterica*, *Campylobacter jejuni*, *Pasteurella multocida*, *Microsporum canis*, flea-borne *Rickettsia* species, and rabies—with emphasis on transmission routes, clinical manifestations, and risk modifiers in children, pregnant women, and immunocompromised individuals. Within a One Health framework, we also summarize global publication trends on feline zoonoses, discuss how urban cat ecology and management (including free-ranging cats in child-frequented environments) may shape pediatric risk, and outline practical prevention strategies centred on hygiene, veterinary care, and targeted education for caregivers and children.

## 1. Introduction

Domestic cats (*Felis catus*) occupy a central role in human households and communities, providing companionship, affection, and emotional support that can alleviate stress and enhance subjective well-being [1]. This attachment often extends beyond privately owned animals to free-ranging “community” cats that people feed and informally care for in public spaces as an expression of empathy, kindness, and community solidarity [2,3]. Municipalities in many regions have responded with policies that tolerate or actively support community feeding and volunteer-run programs, including Trap–Neuter–Return (TNR) for free-roaming cats [3].

At the same time, cats are important reservoirs and vectors for a broad range of zoonotic pathogens, including protozoa, helminths, bacteria, fungi, viruses, and ectoparasite-borne agents, as well as ectoparasites that act as vectors of these [4]. Their pervasive presence across urban, rural, and natural landscapes enables them to bridge ecological interfaces among humans, wildlife, and the environment, facilitating pathogen transmission [5]. Children, pregnant women, and immunocompromised individuals are at particular risk because of biological susceptibility and behavioral exposure patterns [6,7].

Understanding of cat-associated pathogens is a relatively recent development within zoonotic disease research [8,9,10]. Early work in the 1970s and 1980s was limited to sporadic case reports and small surveys on conditions such as leptospirosis and toxoplasmosis, offering only fragmentary insight into the role of domestic cats [11,12]. The 1990s marked a turning point with the rise of molecular epidemiology, including genotyping of *Toxoplasma gondii* and formal identification of *Bartonella henselae* as the etiologic agent of cat scratch disease, which collectively reframed cats as important reservoirs rather than incidental hosts [13].

From 2000 onward, the field matured rapidly. Systematic reviews, global prevalence studies, and meta-analyses on parasites such as *Toxocara* and *Leptospira* and on vector-borne agents in cats began to consolidate scattered primary studies [14]. Between 2020 and 2024, research activity reached an unprecedented peak—approximately 3000 publications over five years—driven by pandemic-associated One Health initiatives, heightened interest in SARS-CoV-2 and avian influenza in cats, and multi-pathogen surveillance of urban stray populations ([15,16,17,18] Table 1).

These historical and bibliometric trends highlight that domestic cats are now recognized as key actors in zoonotic disease systems, rather than peripheral companions. This shift has occurred in parallel with the wider adoption of the One Health concept, which emphasizes that human health, animal health, and environmental conditions are tightly interconnected. The present review builds on this perspective to examine how cat-associated pathogens specifically affect children and other vulnerable groups living in increasingly urbanized, cat-rich environments.

In this review, the focus is on children, pregnant women, and immunocompromised individuals because these groups either experience higher exposure to cats or face more severe outcomes once infected. Children have frequent close contact with pets, use ground-level environments such as sandboxes and playgrounds, and have developing hygiene behaviors that increase their risk of ingesting or contacting infectious stages. Pregnant women and immunocompromised patients, by contrast, are vulnerable to severe sequelae such as congenital toxoplasmosis, disseminated bartonellosis, or fulminant bacterial and fungal infections, even when exposure doses are low. The objectives of this review are threefold: (i) to synthesize scattered evidence on major cat-associated zoonoses that are most relevant for these vulnerable groups; (ii) to interpret these risks in a One Health framework that links human behavior, cat ecology (owned versus free-ranging), and environmental contamination; and (iii) to identify key research and policy gaps, including the scarcity of age-stratified incidence data by exposure route, the limited evaluation of cat-management interventions on human infection endpoints, and the lack of integrated human–animal–environment surveillance, in order to guide future work aimed at protecting vulnerable groups while enabling sustainable human–cat coexistence [9,10,11,12,13,14,15,16,17,18,19,20,21,22,23,24].

## 2. Methods

**Figure 1:**
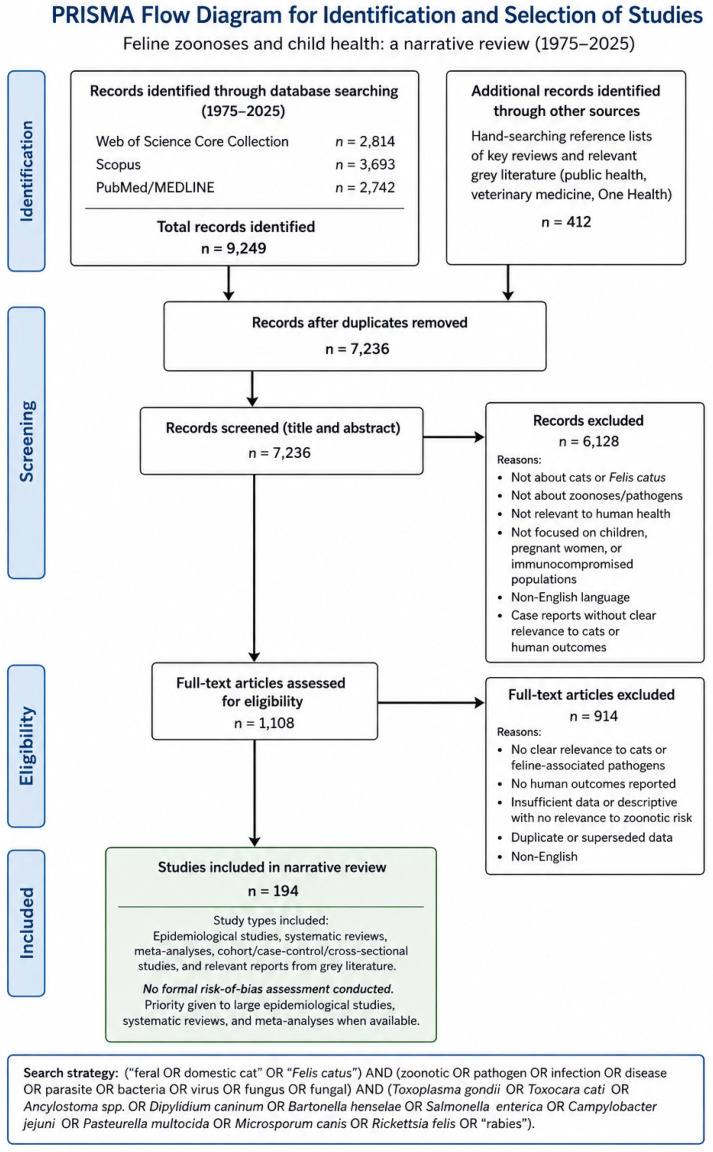
PRISMA flow diagram summarizing the identification, screening, eligibility, and inclusion of studies for this narrative review of zoonotic pathogens associated with domestic cats and their impact on children, showing the number of records retrieved from each database, records excluded at each stage, and the final set of articles included in the synthesis.

This narrative review was conducted following core principles of the Preferred Reporting Items for Systematic Reviews and Meta-Analyses (PRISMA) framework for systematic reviews, adapted to the broad, multidisciplinary scope of feline zoonoses and child health (Figure 1). Web of Science Core Collection, Scopus, and PubMed/MEDLINE were searched for peer-reviewed articles published between 1975 and 2025 using combinations of the terms “feral OR domestic cat” OR “*Felis catus*” AND “zoonotic OR pathogen OR infection OR disease OR parasite OR bacteria OR virus OR fungus OR fungal” together with names of major cat-associated pathogens (e.g., *Toxoplasma gondii*, *Toxocara cati*, *Ancylostoma* spp., *Dipylidium caninum*, *Bartonella henselae*, *Salmonella enterica*, *Campylobacter jejuni*, *Pasteurella multocida*, *Microsporum canis*, *Rickettsia felis*, “rabies”). Titles and abstracts were screened for relevance to zoonotic risk, with particular emphasis on children, pregnant women, and immunocompromised populations; non-English articles and case reports without clear relevance to cats or human outcomes were excluded. Reference lists of key reviews were hand-searched to identify additional studies and grey literature sources relevant to public health, veterinary medicine, and One Health. Because the goal was to provide an integrative overview rather than a quantitative meta-analysis, formal risk-of-bias scoring was not applied; instead, large epidemiological studies, systematic reviews, and meta-analyses were used when available. Because robust pathogen-specific meta-analyses already exist for several of these agents (e.g., *Toxocara* seroprevalence, *Bartonella* in cats), primary data were not re-analyzed; instead, key quantitative estimates from those studies were summarized.

Understanding of cat-associated pathogens is a relatively recent development within zoonotic disease research, emerging alongside broader concern over cross-species pathogen flow and the need for consistent surveillance in high-density urban cat populations [25,26]. Early work in the 1970s and 1980s was limited to sporadic case reports on conditions such as leptospirosis and toxoplasmosis, offering only fragmentary insight into feline zoonotic roles [6]. The 1990s marked a turning point with the rise of molecular epidemiology, including genotyping of *Toxoplasma gondii* and formal identification of *Bartonella henselae* as the etiologic agent of cat scratch disease, which collectively reframed cats as important reservoirs rather than incidental hosts [27]. From 2000 onward, the field matured rapidly, with systematic reviews and global meta-analyses consolidating scattered primary studies into a coherent understanding of feline zoonoses and their public health implications [28,29]. Between 2020 and 2024, research activity reached an unprecedented peak, approximately 3000 publications over five years, driven by pandemic-associated One Health initiatives, heightened interest in SARS-CoV-2 and other cross-species transmission events, and large-scale meta-analyses of vector-borne pathogens in cats (Table 1) [26,30].

The estimated number of publications per pentade was derived by sampling multiple scholarly search engines and databases to approximate temporal trends across disciplines. Specifically, multidisciplinary bibliographic databases such as Web of Science Core Collection, Scopus, and PubMed/MEDLINE were queried using combinations of terms including “feral/domestic cat*” or “*Felis catus*” together with “zoonotic*,” “pathogen*,” “parasite,” “bacteria,” “virus,” “fungal,” and the names of major cat-associated pathogens (e.g., *Toxoplasma gondii*, *Bartonella henselae*, and *Toxocara cati*), with results restricted to each five-year period. These database outputs were then cross-checked against Google Scholar to capture additional veterinary, ecological, and grey-literature sources not fully indexed in the primary databases, again using time-bounded searches by pentade. Finally, trends were spot-checked against selected journal and publisher platforms with strong coverage in infectious diseases, veterinary medicine, and One Health (e.g., MDPI, Elsevier, Springer Nature) to confirm that the overall trajectory, rather than exact counts, was consistent across indexers.

**Table 1:** Estimated number of publications on zoonotic pathogens in domestic and free-ranging cats by five-year period (1975–2024), illustrating the rapid expansion of feline zoonosis research and the recent acceleration associated with One Health and pandemic-related cross-species studies.

Pentade	Estimated Publications	Key Themes
1975–1979	<10	Early disease reports
1980–1984	~30	*Toxoplasma*, *Campylobacter*, *Bartonella*
1990–1994	~100	Epidemiology and zoonotic recognition
2000–2004	~250	Global reviews and diagnostics
2010–2014	~450	Urban ecology and One Health
2020–2024	~3000	Pandemic-era multi-pathogen research

## 3. Major Zoonotic Pathogens Associated with Domestic Cats

Domestic and free-ranging cats can harbor numerous infectious agents, but not all are equally relevant to human health. In this review the term “major zoonotic pathogens” is used for those agents that meet three criteria: (i) there is clear evidence that cats act as reservoirs, amplifying hosts, or direct sources of human infection; (ii) they cause clinically significant disease in children, pregnant women, or immunocompromised individuals; and (iii) there is sufficient epidemiological information (prevalence, incidence, or burden estimates) to support meaningful synthesis (Table 2). Non-zoonotic feline pathogens (e.g., FIV, FeLV, feline panleukopenia virus) are mentioned briefly only where their immunosuppressive effects may indirectly increase zoonotic risk, but they are not treated as primary outcomes. Domestic and free-ranging cats can harbor several zoonotic agents simultaneously, and molecular studies increasingly report co-infections, particularly in high-density urban colonies [31,32,33]. The main pathogen categories of concern are protozoan and helminth parasites, bacteria, viruses, fungi, and ectoparasites that act as vectors for these agents [34,35].

## 4. Parasitic Infections

From a child-health perspective, several pathogens stand out. Congenital toxoplasmosis is estimated to affect between 190,100 and 341,700 infants worldwide each year, with a high proportion developing long-term neurological or ocular impairment. A large meta-analysis of 250 studies (265,327 participants from 71 countries) estimated a global human *Toxocara* seroprevalence of 19.0% (95% CI: 16.6–21.4%), with especially high levels in the African (37.7%) and Southeast Asian (34.1%) WHO regions and lower levels in Europe (10.5%) and the Eastern Mediterranean (8.2%) [47]. Elevated seroprevalence was strongly associated with lower income and human-development indices; lower latitude; warmer, more humid climates; and individual risk factors such as young age, rural residence, close contact with dogs, cats, or soil, consumption of raw meat, and drinking untreated water—all conditions that disproportionately involve or affect children. Ringworm (dermatophytosis) caused by *Microsporum canis* and other zoonotic dermatophytes is another high-frequency outcome of close contact between children and pets; recent compartmental modelling work indicates that sustained transmission in human–pet systems is possible even when clinical disease is self-limiting, highlighting the importance of early diagnosis and treatment in both hosts to reduce recurrent household outbreaks [48]. In the United States, cat scratch disease accounts for roughly 12,000 cases per year, with incidence highest among children aged 5–9 years, while cat-associated rabies, although rarer than dog-mediated rabies, has been increasingly documented in several endemic regions and is invariably fatal without timely post-exposure prophylaxis. Together, these data show that cat-associated zoonoses pose a quantitatively meaningful burden on children and other vulnerable groups, particularly in socio-economically disadvantaged and tropical settings.

### 4.1. Toxoplasma gondii

As summarized in Table 2, *Toxoplasma gondii* is one of the major zoonotic parasites associated with domestic cats [6], with particularly severe consequences for fetuses and immunocompromised individuals. Felids are the only known definitive hosts capable of shedding environmentally resistant oocysts in feces, and cats typically acquire infection by hunting infected prey or ingesting oocysts from the environment. Following primary infection, cats may excrete large numbers of oocysts over a short shedding period; these oocysts become infectious within 1–5 days and can persist in soil, water, and on surfaces for prolonged periods, contaminating homes, gardens, and public play areas.

Large epidemiological studies and recent systematic reviews indicate that, at the population level, the primary routes of human *T. gondii* infection are ingestion of tissue cysts in undercooked or cured meat and ingestion of oocysts via contaminated food or water, rather than direct contact with cats. Meta-analyses conducted in different regions consistently identify consumption of undercooked meat, unwashed raw vegetables, and untreated water as the strongest and most reproducible risk factors for human toxoplasmosis, whereas cat ownership or casual contact with cats shows weaker or inconsistent associations once dietary factors are controlled. This pattern reflects the biology of feline infection: most cats shed oocysts only once, over a relatively short window after primary infection, and then develop immunity, so that prolonged high-level environmental contamination is more closely linked to the cumulative contribution of many cats and to environmental conditions than to continuous shedding by individual pets.

In this context, direct exposure to cat feces or contaminated litter should be regarded as a secondary, context-dependent pathway, important mainly for individuals who handle litter boxes or for children playing in sand or soil where cats defecate, particularly in settings with poor hygiene or high densities of free-ranging cats. By contrast, the major public health burden of toxoplasmosis in both adults and children is driven by food- and water-borne exposure, underscoring the need for safe meat-cooking practices and food-hygiene interventions alongside recommendations on litter-box management and sandbox protection. For children specifically, contaminated play environments (sandboxes, gardens) represent an important local risk, but they occur against a background where food-borne exposure remains the dominant driver of infection at the population scale [49,50,51].

At the population level, global seroprevalence studies report *T. gondii* antibodies in 20–80% of cats and wide geographic variation in human seroprevalence, from about 11% in the United States to over 60% in parts of Latin America, Africa, and Europe. These data, together with the severe congenital and opportunistic forms of disease, underpin the classification of *T. gondii* as a “major zoonotic pathogen” in Table 2 and justify its central role in discussions of cat-associated risk for pregnant women and immunocompromised individuals [35].

### 4.2. Intestinal Helminths: Toxocara cati and Ancylostoma spp.

Cats commonly carry intestinal roundworms (*Toxocara cati*) and hookworms (*Ancylostoma* spp.), which shed eggs or larvae that contaminate soil and other environmental substrates [52]. In humans, ingestion of *Toxocara* eggs can lead to visceral larva migrans (VLM) or ocular larva migrans (OLM), potentially causing hepatic lesions, eosinophilia, or permanent ocular damage. Hookworm larvae (*Ancylostoma* spp.) can penetrate the skin, causing cutaneous larva migrans, characterized by serpiginous, intensely pruritic tracks, often on the feet or buttocks of children playing barefoot in sand or dirt [36,53]. Other intestinal helminths occasionally reported in cats and relevant to humans include cestodes such as *Taenia taeniaeformis* and the zoonotic roundworm *Strongyloides stercoralis*, although their public health impact appears considerably lower than that of *Toxocara* and *Ancylostoma* in most settings. These additional parasites highlight that the intestinal helminth fauna of cats is diverse, but current evidence indicates that *Toxocara cati* and hookworms remain the principal pediatric concerns due to their high prevalence and well-documented roles in larva migrans syndromes.

Children are particularly susceptible because of frequent hand-to-mouth behavior, poor hand hygiene, and ground-level play in contaminated areas [54]. Minimizing environmental contamination through regular deworming of cats, restricting defecation in play areas, and educating caregivers about hygiene is crucial [55].

### 4.3. Fleas, Tapeworms, and Other Vector-Borne Parasites

Cat fleas (*Ctenocephalides felis*) are ubiquitous on domestic and free-ranging cats and can transmit several zoonotic agents. *Dipylidium caninum*, a tapeworm, uses fleas as intermediate hosts; children may become infected by accidentally ingesting an infected flea, resulting in mild gastrointestinal symptoms or the visible passage of proglottids [56]. Fleas also transmit *Bartonella henselae* between cats and may act as vectors of infection to humans, while flea bites themselves can cause allergic dermatitis in both cats and people [57]. A recent global meta-analysis of 131 studies found that *Bartonella* infections are common in companion animals, with pooled prevalence estimates of 15.3% in cats and 3.6% in dogs, particularly in young, free-roaming, flea-infested cats in specific latitude bands. *Bartonella henselae* predominated in cats and *B. vinsonii* subsp. *berkhoffii*, *B. henselae*, *Candidatus B. merieuxii*, and *B. rochalimae* in dogs, underscoring the need for targeted preventive and control measures worldwide [58].

Further, cat fleas can carry *Rickettsia felis*, the agent of flea-borne spotted fever [59], and occasionally *Rickettsia typhi*, contributing to murine typhus cycles in some regions [4]. Effective flea control on cats and in domestic environments is therefore a cornerstone of preventing several cat-associated zoonoses. In addition to fleas and ticks, mites such as *Notoedres* spp. can occasionally cause self-limited zoonotic dermatitis in humans in close contact with infested cats, but current evidence suggests that their overall public-health importance is low compared with the major pathogens summarized in Table 2.

## 5. Bacterial Infections

### 5.1. Bartonella henselae and Cat Scratch Disease

As indicated in Table 2, *Bartonella henselae* is the principal cause of cat scratch disease (CSD) and a major bacterial zoonosis associated with domestic cats, with a disproportionate impact on children and immunocompromised hosts [27]. Cats, especially young ones, may be chronically bacteremic without clinical signs, and the organism is transmitted between cats primarily by cat fleas (*Ctenocephalides felis*). Humans are typically infected via scratches or bites from infected cats or via contamination of broken skin with flea feces.

Clinically, CSD usually presents as a papule or pustule at the inoculation site followed by regional lymphadenopathy, fever, and malaise; in immunocompetent individuals, it is often self-limiting. However, as summarized in Table 2, children are the age group most frequently affected, reflecting close physical interaction, rough play, and limited awareness of safe handling [60,61]. In immunocompromised patients—including those with HIV infection, solid organ transplants, or chemotherapy—*B. henselae* can cause severe disseminated disease such as bacillary angiomatosis, peliosis hepatis, and neuroretinitis.

A recent global meta-analysis of 131 studies involving more than 20,000 cats and nearly 10,000 dogs estimated pooled *Bartonella* prevalence rates of 15.3% in cats and 3.6% in dogs, with higher prevalence in young, free-roaming, flea-infested cats in specific latitude bands [27]. These findings underscore the importance of flea control and bite- and scratch-prevention, particularly in households with children and immunocompromised persons, and support the designation of *B. henselae* as a major cat-associated pathogen of pediatric and One Health concern (Table 2).

### 5.2. Pasteurella multocida and Other Bite-Associated Enteric Bacteria

In addition to *Bartonella henselae*, cats can carry a range of bacterial species that pose direct or indirect risks to humans. *Pasteurella multocida* is a common component of feline oral flora and a frequent cause of rapidly progressive soft-tissue infection following bites or deep scratches, especially in elderly or immunocompromised patients [62]. Enteric pathogens such as *Salmonella enterica*, *Campylobacter jejuni*, and, less commonly, *Listeria monocytogenes* have been isolated from the feces of healthy and diarrheic cats, raising concerns about environmental contamination of households, sandboxes, and gardens used by children. Although the absolute risk to individual owners is low, these organisms can cause severe gastroenteritis, invasive disease, and, in the case of *Listeria*, serious pregnancy-related complications, underscoring the need for good hygiene and food-handling practices in homes with cats.

Cats can also transmit other bacteria, such as *Capnocytophaga canimorsus*, *Staphylococcus aureus* (including methicillin-resistant strains), *Salmonella enterica*, and *Campylobacter jejuni*, contributing to wound infections or gastrointestinal disease [63]. These infections underscore the importance of proper bite and scratch management and avoidance of high-risk interactions.

### 5.3. Rickettsial Agents

Cats and their fleas can participate in the ecology of rickettsial diseases. Fleas on cats have been shown to harbor *Rickettsia felis* and, in some areas, *Rickettsia typhi*, which can cause febrile illnesses such as flea-borne spotted fever and murine typhus in humans [4,43,44,64]. Clinical presentation may include fever, headache, rash, and, occasionally, severe complications if diagnosis and treatment are delayed. Urban free-ranging cats thus contribute to the maintenance of rickettsial cycles in human-dominated environments.

## 6. Viral and Fungal Infections

### 6.1. Viral Infections

Compared with parasites and bacteria, relatively few feline viruses are truly zoonotic. The major exception is the rabies virus, which remains one of the most severe zoonoses globally and can be transmitted by cats as spillover hosts where vaccination coverage is incomplete [4]. Although dogs are responsible for most human rabies cases worldwide, reports of rabid cats and cat-to-human transmission are increasing in some regions, highlighting the importance of routine feline vaccination and post-exposure prophylaxis when cat bites occur [65,66].

By contrast, feline immunodeficiency virus (FIV), feline leukemia virus (FeLV), feline panleukopenia virus (FPLV), and feline upper respiratory viruses (“cat flu”) are not zoonotic but are epidemiologically relevant because they cause immunosuppression and systemic illness in cats. Immunocompromised cats may shed higher loads of enteric bacteria and parasites and are more susceptible to chronic infections, thereby increasing the likelihood of environmental contamination and sustained household transmission cycles; accordingly, we mention these viruses only insofar as they modify zoonotic risk rather than as direct pathogens of humans.

### 6.2. Fungal Infections

Ringworm (dermatophytosis), caused predominantly by *Microsporum canis* in cats, is one of the most frequent zoonotic fungal infections worldwide. Transmission occurs through direct contact with infected animals or contaminated fomites such as bedding, grooming tools, or household surfaces. In humans, especially children, ringworm causes pruritic, circular, scaly lesions on the skin or scalp and can spread readily within households [67].

Less commonly, cats can be involved in the transmission of fungal pathogens such as *Sporothrix schenckii* (sporotrichosis) and *Histoplasma capsulatum* (histoplasmosis) through scratches, bites, or environmental contamination [68,69]. While these infections usually reflect broader environmental exposure, feline involvement in certain endemic areas highlights the need for awareness among clinicians and veterinarians.

## 7. Transmission Routes and Specific Risks for Children

Zoonotic infections associated with cats are transmitted through several key routes that often intersect in everyday family life. The first is direct contact, where scratches, bites, and licks over broken skin or mucous membranes introduce bacteria such as *Bartonella henselae* and *Pasteurella multocida* directly into tissues [27,70]. Children are at elevated risk of this mode of transmission because they frequently engage in rough play, may mishandle cats, and are less likely to recognize or report minor injuries promptly. A second major route is environmental contamination [7,71]. Cat feces can contaminate soil, sandboxes, gardens, and household surfaces with *Toxoplasma gondii* oocysts, *Toxocara* eggs, and hookworm larvae, exposing children through hand-to-mouth behaviour and ground-level play in these areas [72,73]. This kind of indirect exposure is particularly important in communities with high densities of free-ranging cats and limited sanitation or supervision of play spaces.

A third route involves vectors and ectoparasites, principally fleas and ticks. Fleas on cats can bite humans and transmit *Bartonella* and *Rickettsia* species, and they act as intermediate hosts for the tapeworm *Dipylidium caninum* when accidentally ingested by children. Ticks acquired by cats outdoors may also carry pathogens such as *Anaplasma*, *Ehrlichia*, and *Borrelia*, potentially bridging outdoor cycles and peri-domestic environments. Finally, host susceptibility strongly modulates clinical impact. Immature immune systems in children, pregnancy-related immunologic changes, and underlying immune compromise (e.g., HIV infection, chemotherapy, and organ transplantation) all increase the likelihood of severe or complicated disease following exposure to the same pathogens [6,74]. In practical terms, most cat-associated infections in healthy children remain mild or self-limited, but these same agents can cause congenital disease, vision loss, or life-threatening systemic illness in fetuses and immunocompromised individuals, underscoring the need for tailored prevention and counselling in these vulnerable groups [24,75].

## 8. Epidemiological Trends and One Health Perspectives (1975–2025)

A central aim of this review is not only to list pathogens but also to situate cat-associated infections in a One Health context, where urban design, wildlife interfaces, human behavior (e.g., child play patterns, litter-box practices, feeding stations), and veterinary care jointly shape zoonotic risk. By collating evidence across disciplines, this review highlights where current practice insufficiently accounts for children and other vulnerable groups and where integrated surveillance and management could most effectively reduce disease burden. Over the past fifty years, research on zoonotic pathogens in domestic and free-ranging cats has expanded substantially, shifting from isolated case reports to large-scale epidemiological studies and meta-analyses [10,11,12,13,14,15,16,17,18].

From 2000 onward, an increasing number of global prevalence studies, systematic reviews, and meta-analyses appeared, covering parasites such as *Toxocara* and *Leptospira*, and vector-borne agents, as well as integrated One Health analyses that explicitly considered the roles of environment, wildlife, and human behavior [26,29,30,76]. Between 2020 and 2024, annual publication rates on feline-associated zoonoses were estimated to exceed 600–700 per year, driven in part by interest in SARS-CoV-2 exposure in cats, avian influenza spillover, and multi-pathogen surveillance in urban stray cat populations [77,78]. These trends underscore the growing recognition of domestic cats in zoonotic disease ecology and the value of a One Health approach that links the health of humans, animals, and ecosystems [4].

## 9. Urban Cat Ecology and Implications for Child Health

A few well-documented municipal programs illustrate these links. Long-term TNR initiatives in Rishon LeZion (Israel), Rome (Italy), and Córdoba (Spain) have achieved high sterilization coverage and documented stabilization or reduction of free-ranging cat populations, as well as decreases in some infection markers such as *T. gondii* seroprevalence in colony cats [20,21,22,24]. Although these interventions were not designed primarily as pediatric public health programs, they demonstrate that humane, non-lethal population management can be integrated into broader One Health strategies that aim to reduce environmental contamination, bite and scratch incidents, and the density of high-risk cat populations in spaces shared with children.

## 10. Urban Cat Management

Free-ranging cats—stray, abandoned, or feral—make up a substantial proportion of the global *Felis catus* population and often reach high densities in urban spaces heavily used by children, such as parks, playgrounds, schoolyards, and residential courtyards. In these settings, large unmanaged colonies can increase environmental contamination with feces and ectoparasites, thereby amplifying exposure to *Toxoplasma gondii*, *Toxocara cati*, *Ancylostoma* spp., *Bartonella henselae*, and flea-borne *Rickettsia* species. Consequently, strategies that reduce the size and turnover of free-ranging cat populations or stabilize colonies in ways that facilitate vaccination and parasite control have potential downstream benefits for pediatric zoonotic risk, even when infection outcomes have not been measured directly.

Trap–Neuter–Return (TNR) has emerged as the predominant non-lethal management approach in many cities, reflecting both legal protections for cats and public opposition to culling. In a TNR framework, cats are humanely trapped, surgically sterilized, vaccinated, and returned to their original locations, where they may continue to be monitored and, in some cases, receive routine veterinary care [79,80,81]. When implemented at high coverage and maintained over time, TNR programs in Mediterranean and temperate cities such as Rishon LeZion (Israel), Rome (Italy), and Córdoba (Spain) have achieved stabilization or reduction of free-ranging cat numbers and, in Rome, a documented decline in *T. gondii* seroprevalence in colony cats [82]. Although these initiatives were driven primarily by animal welfare and nuisance concerns rather than explicit child health objectives, they illustrate how urban cat management can be integrated into broader One Health strategies: reducing the density of unmanaged cats in child-frequented environments, facilitating systematic vaccination against rabies and other feline pathogens, and improving access to deworming and flea control at the colony level. From a public health standpoint, such integrated programs are best viewed as complementary to, rather than replacements for, household-level interventions such as hygiene promotion, litter-box management, and education of caregivers and children about safe interactions with cats.

## 11. Prevention, Education, and Risk Reduction

Mitigating cat-associated zoonotic risk requires coordinated interventions at the levels of the cat, the environment, and human behavior:

- Routine veterinary care: Regular vaccination, deworming, and flea/tick control for pet cats reduces the prevalence and shedding of many pathogens [52].
- Hygiene and litter management: Changing litter daily and wearing gloves and washing hands thoroughly after handling cats or cleaning litter boxes minimizes exposure to *T. gondii* and enteric bacteria [6].
- Child safety and education: Teaching children safe handling of cats, discouraging rough play, and reinforcing hand-washing after animal contact or outdoor play reduces the risk of bites, scratches, and ingestion of contaminated material [75].
- Clinical red flags after sandbox exposure: If a child has eaten sand or visible fecal material and later develops fever, persistent cough, abdominal pain, eye pain or visual changes, seizures, or prolonged gastrointestinal symptoms, medical evaluation is recommended, with mention of sandbox exposure so clinicians can consider parasitic infections [83].
- Environmental management: Covering sandboxes, discouraging cats from defecating in children’s play areas, and maintaining clean public spaces reduces environmental contamination by parasitic stages [84].
- Population control: Sustained, high-coverage TNR programs, coupled with health checks, vaccination, and parasite control, address the root driver of unmanaged cat populations and their associated zoonotic burden [20,22,24].
- Tailored guidance for vulnerable groups: Pregnant women and immunocompromised individuals should receive specific advice on minimizing exposure to cat feces and avoiding contact with stray or sick cats [6].

These measures are most effective when implemented under a One Health framework that integrates public health services, veterinary authorities, environmental management, and community stakeholders [4].

## 12. Research Gaps

Despite the growing body of evidence, several important research gaps remain. First, for most cat-associated pathogens, there is a lack of child-specific incidence and burden estimates that are stratified by exposure route (e.g., litter-box contact, sandbox use, direct bites and scratches), which limits quantitative risk assessment and targeted guidance for caregivers [85,86]. Second, very few studies formally evaluate how urban cat-management interventions (such as TNR, colony feeding regulation, or mandatory vaccination and deworming) affect infection endpoints in humans, particularly children, rather than only cat population metrics [87]. Third, there is limited integration of One Health surveillance systems that jointly track human cases, veterinary diagnoses, and environmental contamination, making it difficult to identify hotspots where cat-associated zoonoses most threaten vulnerable groups.

## 13. Conclusions

Domestic cats are deeply integrated into human societies and deliver substantial emotional and social benefits but also constitute an important reservoir of zoonotic pathogens, with particular implications for children and other vulnerable populations [1,2,70]. Over the last five decades, the expanding body of research on feline-associated zoonoses has highlighted the multiple pathways through which cats, humans, and the environment interact in urban and rural ecosystems [4,26].

Evidence from diverse regions indicates that humane, non-lethal management of free-ranging cats through TNR, combined with responsible pet ownership, environmental hygiene, and targeted education, can mitigate zoonotic risks while upholding animal welfare and legal protections [20,22,24]. Sustainable coexistence between humans and cats is achievable but depends on coordinated, evidence-based interventions that address both human and animal health within a One Health framework.

## Figures and Tables

**Table 2:** Major zoonotic pathogens associated with domestic cats, with emphasis on children and other vulnerable groups.

Pathogen/Group	Main Human Syndromes	Key Transmission Route(s) from Cats	Particular Relevance for Children/Vulnerable Groups	Key References
*Toxoplasma gondii*	Congenital toxoplasmosis, encephalitis in immunocompromised	Ingestion of oocysts from cat-contaminated soil, sandboxes, litter, food, or water; undercooked meat	Severe congenital disease in infants of mothers infected during pregnancy; severe CNS disease in immunocompromised hosts	[24,27,28,29]
*Toxocara cati*	Visceral and ocular larva migrans	Ingestion of embryonated eggs from contaminated soil, sand, or surfaces	Disproportionate burden in young children using sandboxes/playgrounds; risk of permanent visual loss	[25,30,31,32]
*Ancylostoma* spp.	Cutaneous larva migrans	Skin penetration by infective larvae in contaminated sand/soil	Common in children playing barefoot; intense pruritus and secondary infections	[36]
*Dipylidium caninum*	Mild GI symptoms, anal pruritus	Ingestion of infected fleas from cats	Mostly affects young children; highlights importance of flea control	[37,38]
*Bartonella henselae*	Cat scratch disease, bacillary angiomatosis (immunocompromised)	Scratches, bites, contamination of broken skin with flea feces	Highest CSD incidence in children 5–9 years; severe disseminated disease in immunocompromised	[39,40]
*Salmonella enterica*, *Campylobacter jejuni*, *Listeria monocytogenes*	Gastroenteritis, invasive infections, pregnancy complications (*Listeria*)	Fecal contamination of environment, hands, or food; sometimes bites/scratches	Children particularly susceptible to dehydration and complications; pregnancy risk for *Listeria*	[41,42]
*Microsporum canis* and other dermatophytes	Ringworm (dermatophytosis) of skin and scalp	Direct contact with infected cats or contaminated fomites	One of the most common pet-associated zoonoses in children; recurrent household outbreaks	[26,43,44,45]
Flea-borne *Rickettsia felis*, *R. typhi*	Flea-borne spotted fever, murine typhus	Bites of infected fleas from cats	Febrile illness, sometimes severe; exposure linked to flea-infested cats in crowded urban settings	[46,47]
Rabies virus	Acute, almost invariably fatal encephalitis	Bites or saliva contact from rabid cats (spillover from wildlife or dogs)	Even rare events have catastrophic outcomes; children at particular risk of under-reported bites and delayed post-exposure prophylaxis	[43,44]

## Data Availability

No original data were created.

## Abbreviations

The following abbreviations are used in this manuscript:

MDPI	Multidisciplinary Digital Publishing Institute
TNR	Trap–Neuter–Return
HIV/AIDS	Human Immunodeficiency Viruses
VLM	Visceral Larva Migrans
OLM	Ocular Larva Migrans
CSD	Cat Scratch Disease
FIV	Feline Immunodeficiency Virus
FeLV	Feline Leukemia Virus

## References

[1] Crawford C., Rand J., Rohlf V., Scotney R., Bennett P. (2023). Solutions-Based Approach to Urban Cat Management-Case Studies of a One Welfare Approach to Urban Cat Management. Animals.

[2] Dillenseger C., Maloutas T., Spyrellis S. (2023). Stray cats between urban planning, heritagization and ecofeminism. Digital Compendium of Texts and Visual Material.

[3] Wolf P.J., Schaffner J.E. (2019). The Road to TNR: Examining Trap-Neuter-Return Through the Lens of Our Evolving Ethics. Front. Vet. Sci..

[4] Gerhold R.W., Jessup D.A. (2013). Zoonotic diseases associated with free roaming cats. Zoonoses Public Health.

[5] Sumi A., Toyoda S., Kanou K., Fujimoto T., Mise K., Kohei Y., Koyama A., Kobayashi N. (2017). Association between meteorological factors and reported cases of hand, foot, and mouth disease from 2000 to 2015 in Japan. Epidemiol. Infect..

[6] Montoya J.G., Rosso F. (2005). Diagnosis and management of toxoplasmosis. Clin. Perinatol..

[7] Ismael S.S., Hasan S.H. (2023). What can you get from your Cat?. Biol. Times Mag..

[8] Szentivanyi T., Oedin M., Rocha R. (2024). Cat–wildlife interactions and zoonotic disease risk: A call for more and better community science data. Mammal Rev..

[9] Chomel B.B. (2014). Emerging and re-emerging zoonoses of dogs and cats. Animals.

[10] Diakou A., Di Cesare A., Accettura P.M., Barros L., Iorio R., Paoletti B., Frangipane di Regalbono A., Halos L., Beugnet F., Traversa D. (2017). Intestinal parasites and vector-borne pathogens in stray and free-roaming cats living in continental and insular Greece. PLoS Negl. Trop. Dis..

[11] Sykes J.E., Reagan K.L., Nally J.E., Galloway R.L., Haake D.A. (2022). Role of diagnostics in epidemiology, management, surveillance, and control of leptospirosis. Pathogens.

[12] Ratti G. (2024). Feline Infectious Diseases: Genetic Characterization of Emerging and Zoonotic Pathogens. Ph.D. Thesis.

[13] Foley J.E., Sellon R.K., Mills W.Z., Evermann J.F., Sykes J.E., Rankin S.C. (2021). Clinical Epidemiology in Infectious Diseases and Interpretation of Diagnostic Tests. Greene’s Infectious Diseases of the Dog and Cat.

[14] Sanyaolu A. (2016). Epidemiology of zoonotic diseases in the United States: A comprehensive review. J. Infect. Dis. Epidemiol..

[15] Melidou A., Enkirch T., Willgert K., Adlhoch C., Alm E., Lamb F., Marangon S., Monne I., Stegeman J.A. (2024). Drivers for a pandemic due to avian influenza and options for One Health mitigation measures. EFSA J..

[16] Bruno L., Nappo M.A., Frontoso R., Montinaro S., Di Lecce R., Guarnieri C., Ferrari L., Corradi A. (2026). Avian Influenza Viruses: Global Panzootic, Host Range Expansion and Emerging One-Health Threats. Vet. Sci..

[17] Marchi S., Guarducci G., Marotta M.G., Peccetti B., Viviani S., Messina G., Montomoli E., Martella V., Camero M., Trombetta C.M. (2024). Improving the ONE HEALTH approach: A lesson from SARS-CoV-2 pandemic. J. Prev. Med. Hyg..

[18] Sikkema R.S., Koopmans M. (2026). Viral emergence and pandemic preparedness in a One Health framework. Nat. Rev. Microbiol..

[19] Stose L.L., Samples O.M., McCommon G.W., Terrill T.H., Stose L.L. (2025). Cat Scratch Fever (*Bartonella* spp.). The One Health Model as Applied to Zoonotic Diseases.

[20] Natoli E., Maragliano L., Cariola G., Faini A., Bonanni R., Cafazzo S., Fantini C. (2006). Management of feral domestic cats in the urban environment of Rome (Italy). Prev. Vet. Med..

[21] Veronesi F., Santilli A., Laus F., De Martino L. (2019). The effects and contribution of childhood diseases on the geographical distribution of all-cause under-five mortality in Uganda. Parasite Epidemiol. Control.

[22] Gunther I., Finkler H., Gabet A., Shochat E., Klement E., Terkel J. (2022). Reduction of free-roaming cat population requires high-intensity neutering in spatial contiguity to mitigate compensatory effects. Proc. Nat. Acad. Sci. USA.

[23] Lepczyk C.A., Fantle-Lepczyk J.E., Dunham K.D., Bonnaud E., Lindner J., Doherty T.S., Woinarski J.C. (2023). A global synthesis and assessment of free-ranging domestic cat diet. Nat. Commun..

[24] Lozano J., Macías-Díaz J., Calzada J., Virgós E. (2025). Four Years of Promising Trap–Neuter–Return (TNR) in Córdoba, Spain: A Scalable Model for Urban Feline Management. Animals.

[25] Rahman M.T., Sobur M.A., Islam M.S., Ievy S., Hossain M.J., El Zowalaty M.E., Rahman A.T., Ashour H.M. (2020). Zoonotic Diseases: Etiology, Impact, and Control. Microorganisms.

[26] Schäfer I., Kohn B., Volkmann M., Müller E. (2021). Retrospective evaluation of vector-borne pathogens in cats living in Germany (2012–2020). Parasites Vectors.

[27] Chomel B.B., Boulouis H.J., Maruyama S., Breitschwerdt E.B. (2006). *Bartonella* spp. in pets and effect on human health. Emerg. Infect. Dis..

[28] Goldstein E.J.C., Abrahamian F.M., Schlossberg D. (2016). Diseases Transmitted by Cats. Infections of Leisure.

[29] Azócar-Aedo L. (2022). Global prevalence and epidemiology of leptospirosis in domestic cats, a systematic review and meta-analysis. Vet. Méx. OA.

[30] Chothe S.K., Srinivas S., Misra S., Nallipogu N.C., Gilbride E., LaBella L., Mukherjee S., Gauthier C.H., Pecoraro H.L., Webb B.T. (2025). Marked neurotropism and potential adaptation of H5N1 clade 2.3. 4.4. b virus in naturally infected domestic cats. Emerg. Microbes Infect..

[31] Pennisi M.G., Marsilio F., Hartmann K., Lloret A., Addie D., Belák S., Boucraut-Baralon C., Egberink H., Frymus T., Gruffydd-Jones T. (2013). *Bartonella* species infection in cats: ABCD guidelines on prevention and management. J. Feline Med. Surg..

[32] Baneth G., Peri Markovich M., Nachum-Biala Y., Yasur-Landau D., Leszkowicz Mazuz M., Jaffe C.L. (2025). Retrospective study of canine leishmaniosis in Israel. Parasites Vectors.

[33] Pal M., Tolawak D., Garedaghi Y. (2023). A comprehensive review on major zoonotic parasites from dogs and cats. Int. J. Med. Parasitol. Epidemiol. Sci..

[34] Udainiya S., Tiwari A., Mishra A., Dubey A. (2024). Zoonotic diseases of dogs and cats. Introduction to Diseases, Diagnosis, and Management of Dogs and Cats.

[35] Dubey J.P. (2016). Toxoplasmosis of Animals and Humans.

[36] Bowman D.D., Montgomery S.P., Zajac A.M., Eberhard M.L., Kazacos K.R. (2010). Hookworms of dogs and cats as agents of cutaneous larva migrans. Trends Parasitol..

[37] Rousseau J., Castro A., Novo T., Maia C. (2022). *Dipylidium caninum* in the twenty-first century: Epidemiological studies and reported cases in companion animals and humans. Parasites Vectors.

[38] Little S., Braff J., Duncan K., Elsemore D., Hanna R., Hanscom J., Lee A., Martin K.A., Sobotyk C., Starkey L. (2023). Diagnosis of canine intestinal parasites: Improved detection of *Dipylidium caninum* infection through coproantigen testing. Vet. Parasitol..

[39] McShane D.B., Kong H.H., Myers S.A. (2011). Bartonella infections: *Bacillary angiomatosis*, cat scratch disease and bartonellosis. Harper’s Textbook of Pediatric Dermatology.

[40] Homsi Y., Milam E., Dayoub N. (2025). Cutaneous Bacillary Angiomatosis: A Rare and Forgotten Infection in Immunocompromised Patient. Avicenna J. Med..

[41] Chlebicz A., Śliżewska K. (2018). Campylobacteriosis, salmonellosis, yersiniosis, and listeriosis as zoonotic foodborne diseases: A review. Int. J. Environ. Res. Public Health.

[42] Wang Z., Tao X., Liu S., Zhao Y., Yang X. (2021). An update review on Listeria infection in pregnancy. Infect. Drug Resist..

[43] Benavides J.A., Megid J., Campos A., Hampson K. (2020). Using surveillance of animal bite patients to decipher potential risks of rabies exposure from domestic animals and wildlife in Brazil. Front. Public Health.

[44] Tuglo L.S., Branda F., Mohapatra R.K., Pattnaik P., Sahu A.R., Mishra S. (2026). Spill-over rabies transmission–a neglected aspect in african rabies control efforts. Infect. Dis..

[45] Brown L.D., Macaluso K.R. (2016). *Rickettsia felis*, an emerging flea-borne rickettsiosis. Curr. Trop. Med. Rep..

[46] Caravedo Martinez M.A., Ramírez-Hernández A., Blanton L.S. (2021). Manifestations and management of flea-borne rickettsioses. Res. Rep. Trop. Med..

[47] Rostami A., Riahi S.M., Holland C.V., Taghipour A., Khalili-Fomeshi M., Fakhri Y., Omrani V.F., Hotez P.J., Gasser R.B. (2019). Seroprevalence estimates for toxocariasis in people worldwide: A systematic review and meta-analysis. PLoS Negl. Trop. Dis..

[48] Gweryina R.I., Ashezua T.T., Edeh A. (2025). Compartmental modelling for the transmission dynamics of ringworm disease in humans and pets with bifurcation analysis. Discov. Public Health.

[49] Shapiro K., Bahia-Oliveira L., Dixon B., Dumètre A., de Wit L.A., VanWormer E., Villena I. (2019). Environmental transmission of *Toxoplasma gondii*: Oocysts in water, soil and food. Food Waterborne Parasitol..

[50] Zhu S., Shapiro K., VanWormer E. (2022). Dynamics and epidemiology of *Toxoplasma gondii* oocyst shedding in domestic and wild felids. Transbound. Emerg. Dis..

[51] Marín-García P.J., Planas N., Llobat L. (2022). *Toxoplasma gondii* in foods: Prevalence, control, and safety. Foods.

[52] Hill S.L., Cheney J.M., Taton-Allen G.F., Reif J.S., Bruns C., Lappin M.R. (2000). Prevalence of enteric zoonotic organisms in cats. J. Am. Vet. Med. Assoc..

[53] Vetter J.C.M., Leegwater-vd Linden M.E. (1977). Skin penetration of infective hookworm larvae: I. The path of migration of infective larvae of *Ancylostoma braziliense* in canine skin. Z. Parasitenkd..

[54] Xie Y.H., Chongsuvivatwong V., Tan Y., Tang Z.Z., Sornsrivichai V., McNeil E.B. (2015). Important roles of public playgrounds in the transmission of hand, foot, and mouth disease. Epidemiol. Infect..

[55] Kwong L.H., Ercumen A., Pickering A.J., Unicomb L., Davis J., Luby S.P. (2020). Age-related changes to environmental exposure: Variation in the frequency that young children place hands and objects in their mouths. J. Expo. Sci. Environ. Epidemiol..

[56] Clark N.J., Seddon J.M., Šlapeta J., Wells K. (2018). Parasite spread at the domestic animal-wildlife interface: Anthropogenic habitat use, phylogeny and body mass drive risk of cat and dog flea (*Ctenocephalides* spp.) infestation in wild mammals. Parasites Vectors.

[57] Higgins J.A., Radulovic S., Jaworski D.C., Azad A.F. (1996). Acquisition of the cat scratch disease agent *Bartonella henselae* by cat fleas (Siphonaptera: Pulicidae). J. Med. Entomol..

[58] Zarea A.A.K., Tempesta M., Odigie A.E., Mrenoshki D., Fanelli A., Martella V., Decaro N., Greco G. (2023). The Global Molecular Prevalence of *Bartonella* spp. in Cats and Dogs: A Systematic Review and Meta-Analysis. Transbound. Emerg. Dis..

[59] Hirunkanokpun S., Thepparit C., Foil L.D., Macaluso K.R. (2011). Horizontal transmission of *Rickettsia felis* between cat fleas, *Ctenocephalides felis*. Mol. Ecol..

[60] Purewal R., Christley R., Kordas K., Joinson C., Meints K., Gee N., Westgarth C. (2017). Companion animals and child/adolescent development: A systematic review of the evidence. Int. J. Environ. Res. Public Health.

[61] Cuomo C.A. (2021). The relaunch of microbiology spectrum. Microbiol. Spectr..

[62] Wei B., Liu C., Zhu J., Zou X., Zhang Z. (2025). *Pasteurella multocida* infection: A differential retrospective study of 482 cases of *P. multocida* infection in patient of different ages. BMC Infect. Dis..

[63] Beauruelle C., Plouzeau C., Grillon A., Isnard C., Corvec S., Degand N., Jacquier H., Amara M., Mizrahi A., Diedrich T. (2022). *Capnocytophaga* zoonotic infections: A 10-year retrospective study (the French CANCAN study). Eur. J. Clin. Microbiol. Infect. Dis..

[64] Schriefer M.E., Sacci J.B., Dumler J.S., Bullen M.G., Azad A.F. (1994). Identification of a novel rickettsial infection in a patient diagnosed with murine typhus. J. Clin. Microbiol..

[65] Fehlner-Gardiner C., Gongal G., Tenzin T., Sabeta C., De Benedictis P., Rocha S.M., Vargas A., Cediel-Becerra N., Gomez L.C., Maki J. (2024). Rabies in cats—An emerging public health issue. Viruses.

[66] Das M., Yustyniuk V., Perez A.M., Aguirreburualde M.S.P. (2025). Global Perspectives on Rabies Control and Elimination: A Scoping Review of Dog Owners’ Knowledge, Attitudes, and Practices. Pathogens.

[67] Hobi S., Tam W.Y.J., Tse M., Nekouei O., Chai Y., Hill F.I., Cheung E., Botes W., Saulnier-Troff F., McDermott C.T. (2024). *Microsporum canis* Causes Cutaneous and Extracutaneous Feline Dermatophytic Pseudomycetomas: Molecular Identification and Clinicopathological Characteristics. J. Fungi.

[68] Barrs V.R., Bęczkowski P.M., Talbot J.J., Hobi S., Teoh S.N., Hernandez Muguiro D., Shubitz L.F., Sandy J. (2024). Invasive fungal infections and oomycoses in cats: 1. Diagnostic approach. J. Feline Med. Surg..

[69] Eissa N. (2024). Fungal diseases of dogs and cats. Introduction to Diseases, Diagnosis, and Management of Dogs and Cats.

[70] Septelici D., Carbone G., Cipri A., Esposito S. (2024). Management strategies for common animal bites in pediatrics: A narrative review on the latest progress. Microorganisms.

[71] Dixit B., Kumar R., Dixit A.K., Singh A.K., Rana T. (2024). Risk Factors Associated with Parasitic Diseases in Dogs and Cats. Principles and Practices of Canine and Feline Clinical Parasitic Diseases.

[72] Morelli S., Diakou A., Di Cesare A., Colombo M., Traversa D. (2021). Canine and feline parasitology: Analogies, differences, and relevance for human health. Clin. Microbiol. Rev..

[73] Aleem M.T., Munir F., Shakoor A., Rana T. (2024). Parasitic diseases of dogs and cats. Introduction to Diseases, Diagnosis, and Management of Dogs and Cats.

[74] Kumar M., Saadaoui M., Al Khodor S. (2022). Infections and pregnancy: Effects on maternal and child health. Front. Cell. Infect. Microbiol..

[75] Jackson L.A., Perkins B.A., Wenger J.D. (1993). Cat scratch disease in the United States: An analysis of three national databases. Am. J. Public Health.

[76] Andityas M., Nuraini D.M., Sota P., Loong S.K., Sripa B., Sukon P., Tangkawattana P., Tangkawattana S. (2024). Feline leptospirosis prevalence worldwide: A systematic review and meta-analysis of diagnostic approaches. Vet. World.

[77] Schulz C., Martina B., Mirolo M., Müller E., Klein R., Volk H., Egberink H., Gonzalez-Hernandez M., Kaiser F., von Köckritz-Blickwede M. (2021). SARS-CoV-2–specific antibodies in domestic cats during first COVID-19 wave, Europe. Emerg. Infect. Dis..

[78] Coleman K.K., Bemis I.G. (2025). Avian influenza virus infections in felines: A systematic review of two decades of literature. Open Forum Infect. Dis..

[79] Loss S.R., Will T., Marra P.P. (2013). The impact of free ranging domestic cats on wildlife of the United States. Nat. Commun..

[80] Pena H., Soares R., Amaku M., Dubey J., Gennari S. (2006). *Toxoplasma gondii* infection in cats from São Paulo state, Brazil: Seroprevalence, oocyst shedding, isolation in mice, and biologic and molecular characterization. Res. Vet. Sci..

[81] Natoli E. (2024). The intrinsic moral value of individuals: A bioethical approach to domestic cats and damaged species. Appl. Anim. Behav. Sci..

[82] Peris M.P., Planas S., Langa J., Laborda A., Castillo J.A., Gracia M.J. (2024). Seroprevalence of zoonotic pathogens in stray cats in an urban area of northeast Spain. Vet. Parasitol. Reg. Stud. Rep..

[83] Callahan G.N., Whitaker E.D. (2016). Eating dirt. Health and Healing in Comparative Perspective.

[84] Torrey E.F. (2021). Sentinel Seals, Safe Cats, and Better Treatments. Parasites, Pussycats and Psychosis: The Unknown Dangers of Human Toxoplasmosis.

[85] Spindel M., Sykes J.E. (2021). Prevention and management of infectious diseases in multiple-cat environments. Greene’s Infectious Diseases of the Dog and Cat.

[86] Candela M.G., Fanelli A., Carvalho J., Serrano E., Domenech G., Alonso F., Martínez-Carrasco C. (2022). Urban landscape and infection risk in free-roaming cats. Zoonoses Public Health.

[87] Cotterell J., Rand J., Scotney R. (2025). Urban cat Management in Australia—Evidence-based strategies for success. Animals.

